# Protective role for lipid modifications of Src-family kinases against chromosome missegregation

**DOI:** 10.1038/srep38751

**Published:** 2016-12-12

**Authors:** Takuya Honda, Shuhei Soeda, Kunihiko Tsuda, Chihiro Yamaguchi, Kazumasa Aoyama, Takao Morinaga, Ryuzaburo Yuki, Yuji Nakayama, Noritaka Yamaguchi, Naoto Yamaguchi

**Affiliations:** 1 Laboratory of Molecular Cell Biology, Graduate School of Pharmaceutical Sciences, Chiba University, Chiba 260-8675, Japan; 2 Department of Biochemistry and Molecular Biology, Kyoto Pharmaceutical University, Kyoto 607-8414, Japan

## Abstract

Src-family tyrosine kinases, which are expressed in various cell types, play critical roles in cell signalling at the cytoplasmic side of the plasma membrane through their lipid modifications. Src-family kinases are cotranslationally myristoylated and posttranslationally palmitoylated in the amino-terminal region. The Src-family member Lyn contains a myristoylation site at glycine-2 and a palmitoylation site at cysteine-3, whereas c-Src has a myristoylation site at glycine-2 but not any palmitoylation sites. However, little is known about the role for lipid modifications of Src-family kinases in cell division. Here, we show that non-lipid-modified Lyn and c-Src, Lyn(G2A/C3A) and c-Src(G2A), are delocalized from membranes to the cytoplasm and the nucleus, which gives rise to a significant increase in the rate of chromosome missegregation, such as chromosome lagging and anaphase chromosome bridging, in a tyrosine kinase activity-dependent manner. Treatment with the Src inhibitor PP2 shows that the kinase activity of non-lipid-modified, non-membrane-bound Src during M phase is critical for giving rise to chromosome missegregation. Given that only a fraction of Src-family kinases fails in lipid modifications during biosynthesis, these results suggest that Src’s membrane anchorage through their lipid modifications from prophase to anaphase plays a protective role against induction of chromosome missegregation.

Src-family kinases, a family of non-receptor-type tyrosine kinases, are expressed in a variety of organs, tissues and cell types, including epithelial, hematopoietic and neuronal cells, and play critical roles in cell signalling at the cytoplasmic side of the plasma membrane^1^,^2^. Src-family kinases include at least eight highly homologous members: c-Src, Lyn, c-Yes, Fyn, c-Fgr, Hck, Lck, and Blk. They have six major domains: (i) an amino-terminal Src homology (SH) 4 domain that contains lipid modification sites, (ii) a unique domain, (iii) an SH3 domain, which binds to proline-rich sequences, (iv) an SH2 domain that binds to a phosphotyrosine-containing motif, (v) a catalytic domain, and (vi) a carboxyl-terminal negative regulatory region^1^,^2^. In addition, v-Src is an oncogenic mutant of the cellular proto-oncogene c-Src owing to the lack of the carboxyl-terminal negative regulatory tyrosine residue^3^,^4^.

All members of the Src family, in which the initiating methionine is removed by methionine aminopeptidase, are cotranslationally myristoylated at glycine-2 in the amino-terminal SH4 domain and, with the exception of c-Src and Blk, are also posttranslationally palmitoylated at cysteine-3, cysteine-5 or cysteine-6. Lyn, a mono-myristoylated and mono-palmitoylated Src-family kinase, contains glycine-2 for myristoylation and cysteine-3 for palmitoylation in the SH4 domain, both of which are necessary for membrane anchorage of Lyn^5^,^6^. We showed that Lyn is exocytosed toward the plasma membrane via the Golgi region along the secretory pathway following its biosynthesis in the cytoplasm^7^,^8^,^9^,^10^,^11^. In addition, palmitoylation of Lyn, Lck, and Fyn at cysteine-3 is important for their membrane localization and trafficking^5^,^6^,^10^,^12^,^13^,^14^.

Although Src-family kinases have crucial roles in cell signalling at the cytoplasmic side of the plasma membrane^15^,^16^, the roles of Src-mediated tyrosine phosphorylation in the nucleus have not been fully understood. We have shown that at least four members of the Src family, c-Src, Lyn, Fyn and c-Yes, are endogenously present in nuclei of HeLa cells and hematopoietic cells^17^,^18^,^19^,^20^. Recently, we revealed that KRAB-associated protein 1 (KAP1) is tyrosine-phosphorylated by nuclear tyrosine kinases, including c-Src, and that tyrosine phosphorylation of KAP1 inhibits the association of KAP1 and heterochromatin protein 1α (HP1α) with heterochromatin^21^. We also uncovered that tyrosine phosphorylation of A-kinase anchoring protein 8 (AKAP8) by nuclear tyrosine kinases, including c-Src, dissociates AKAP8 from chromatin and the nuclear matrix^22^. These results demonstrate the significance of nucleus-localized Src-family kinases in dynamic chromatin regulation. Furthermore, Src-family kinases have been reported to regulate cell division^23^,^24^,^25^. We then found the roles of Src-family kinases in spindle orientation, spindle assembly, and cytokinesis abscission^17^,^26^,^27^,^28^. Despite the importance of membrane anchorage of Src-family kinases, the role for their lipid modifications in cell division is largely unknown.

In this study, we showed that the inability of Src-family kinases to be modified with lipids is responsible for induction of chromosome missegregation, such as chromosome lagging and anaphase chromosome bridging. We further showed that the tyrosine kinase activity of non-lipid-modified Src-family kinases appearing from early prophase is deeply involved in induction of chromosome missegregation. Our findings raise the intriguing possibility that membrane anchorage of Src-family kinases via lipid modification may protect normal cell division from non-membrane-bound Src-induced chromosome missegregation.

## Results

### Inducible Src-dependent inhibition of M-phase entry

**Figure 1:**
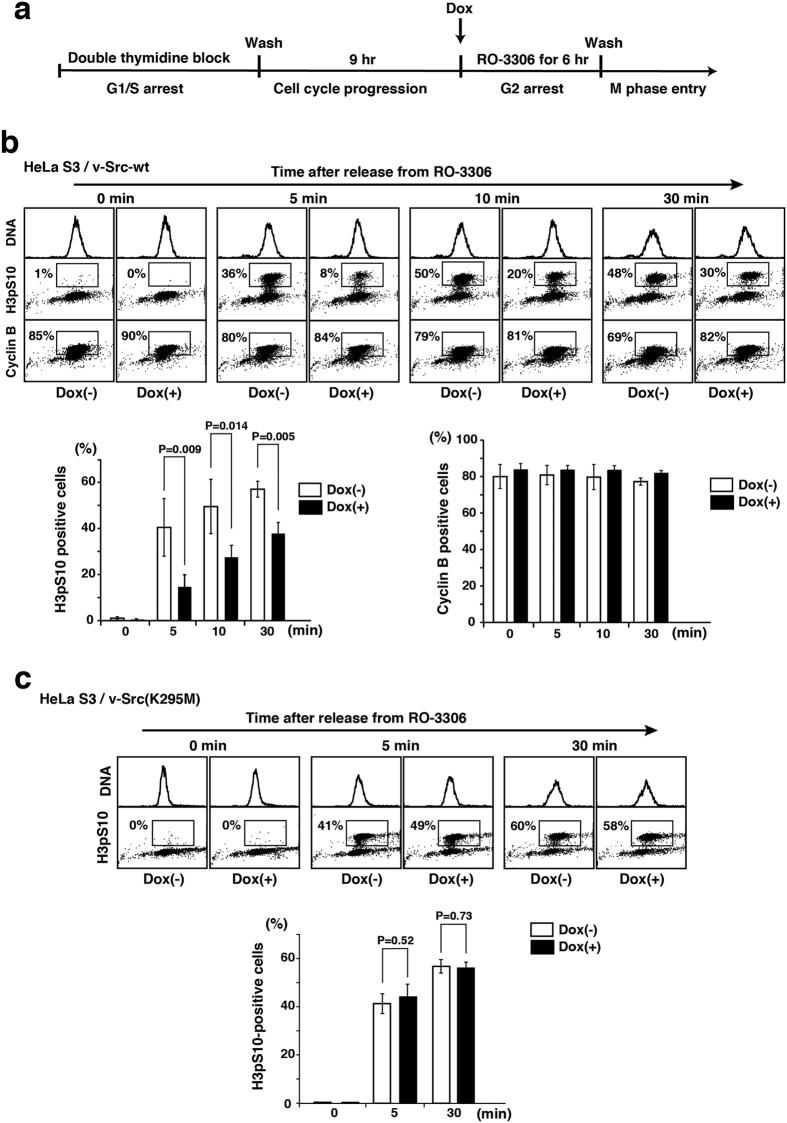
Inhibition of M-phase entry by inducible Src. (**a**) Schematic depiction of cell-cycle synchronization at the onset of M phase. Cells synchronized by double thymidine block were incubated for 9 h in thymidine-free medium, subsequently treated for 6 h with 9 μM RO-3306 in the presence or absence of 1 μg/ml Dox for inducible expression in G2 phase, and released from RO-3306 treatment for the indicated time. (**b**) HeLa S3/TR cells stably expressing inducible v-Src-wt (HeLa S3/v-Src-wt) were triply stained with propidium iodide (DNA staining) and anti-phospho-histone H3 Ser10 (H3pS10) and anti-cyclin B1 antibodies for analyzing M-phase entry by flow cytometry (panels) and quantitating cells entering M phase (H3pS10-positive) and cells proceeding into G2 or M phases (cyclin B1-positive) (graphs). Values are means ± S.D. from three independent experiments, and the significant differences are calculated by Student’s *t*-test. (**c**) HeLa S3/TR cells stably expressing inducible v-Src(K295M) (HeLa S3/v-Src(K295M)) were synchronized by the same method as shown in (**a**) and stained with propidium iodide and anti-H3pS10 antibody for analyzing M-phase entry by flow cytometry (panels) and quantitating M-phase cells (graph). P = 0.52 and P = 0.73 indicate no significant differences calculated by Student’s *t*-test.

Recently, we generated HeLa S3 and HCT116 cell lines stably expressing tetracycline-inducible v-Src (HeLa S3/v-Src and HCT116/v-Src), and showed that v-Src expression inhibits cell proliferation in a kinase activity-dependent manner^29^. Also, we showed that inducible v-Src gives rise to a significant increase in chromosome bridge formation^29^. To analyze whether v-Src expression affected cell cycle progression from G2 phase to M phase, HeLa S3/v-Src cells that were arrested at G1/S phase were further synchronized at the G2/M boundary in the presence of the cyclin-dependent kinase 1 (Cdk1) inhibitor RO-3306, and v-Src was induced by the addition of doxycycline (Dox) in G2 phase. After release from cell cycle arrest at the G2/M boundary, cells were analyzed for cyclin B1 expression (a G2 and M phase marker) and phosphorylation of histone H3 at serine-10 (H3pS10, a mitotic marker) (Fig. 1a). Flow cytometric analysis showed that v-Src expression in G2 phase decreased the number of M-phase cells, thereby giving rise to inhibition of the onset of M phase (Fig. 1b). However, inducible expression of a kinase-dead v-Src mutant [v-Src(K295M)] did not affect the onset of M phase (Fig. 1c). Considering that activation of c-Src is found during M phase^30^,^31^, these results suggest that expression of v-Src during G2 and M phases profoundly affects the onset of M phase in a kinase activity-dependent manner, which may link to chromosome missegregation.

### Induction of chromosome missegregation by NLS-Lyn expression

**Figure 2:**
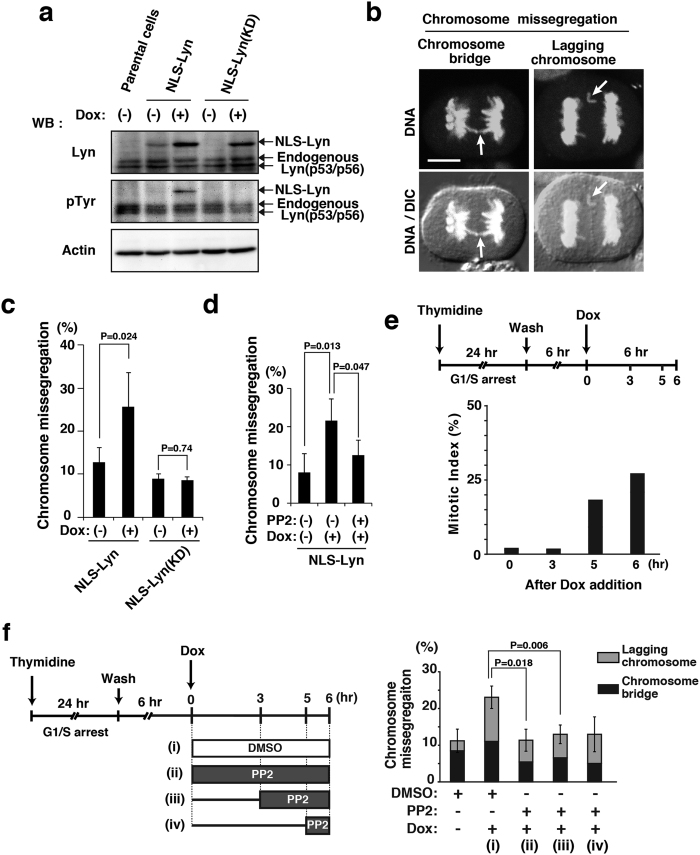
Chromosome missegregation induced by NLS-Lyn. HeLa S3/TR cells stably expressing inducible NLS-Lyn (HeLa S3/NLS-Lyn cells) or inducible NLS-Lyn(KD) (HeLa S3/NLS-Lyn(KD) cells) and HeLa S3/TR parental cells were arrested at G1/S phase by 24 h-thymidine treatment, subsequently released into fresh medium for 12 h, and incubated with 1 μg/ml Dox during the last 6 h (see **e**). (**a**) Cells were arrested at G1/S phase, released for 6 h, and incubated for 6 h in medium with or without Dox. Whole cell lysates from HeLa S3/NLS-Lyn, HeLa S3/NLS-Lyn(KD), and vector-transfected HeLa S3/TR parental cells were analyzed by Western blotting using anti-Lyn, anti-phosphotyrosine (pTyr), and anti-actin (loading control) antibodies. Full-length blots are presented in Supplementary Fig. S1. (**b**) Dox-treated HeLa S3/NLS-Lyn cells were stained for DNA. Arrows indicate an anaphase bridge and a lagging chromosome (phenotypes of chromosome missegregation). Scale bar, 10 μm. (**c**) HeLa S3/NLS-Lyn cells and HeLa S3/NLS-Lyn(KD) cells were treated with Dox for 6 h (see **e**). The number of cells exhibiting chromosome missegregation was counted. Values are means ± S.D. from more than three independent experiments [HeLaS3/NLS-Lyn (>483 cells), HeLa S3/NLS-Lyn(KD) (>516 cells)], and the significant difference is calculated by the Student’s *t*-test. P = 0.74 indicates no significant difference. (**d**) HeLa S3/NLS-Lyn cells were treated with 10 μM PP2 for the last 3 h during Dox treatment (see **f**). The number of cells exhibiting chromosome missegregation was counted. Values are means ± S.D. from more than three independent experiments [HeLa S3/NLS-Lyn (>426 cells)], and the significant differences are calculated by the Student’s *t*-test. (**e**) Schematic depiction of cell synchronization and PP2 treatment. The appearance of mitotic cells was examined after Dox addition by counting the number of H3pS10-positive HeLa S3/NLS-Lyn cells (mitotic index). (**f**) Schematic depiction of cell synchronization and stepwise PP2 addition. HeLa S3/NLS-Lyn cells were treated with or without 10 μM PP2 for the indicated times. The number of cells exhibiting chromosome missegregation was counted. Significant differences are calculated by the Student’s *t*-test.

v-Src, the viral oncogene product, lacks the negative regulatory tyrosine residue at the carboxyl-terminus and is a constitutively active form of its cellular counterpart c-Src^3^. We have shown that Src-family tyrosine kinases play important roles in mitosis^14^,^18^,^26^,^27^,^28^,^32^. In particular, mitotic activation of c-Src, c-Yes, Lyn and Fyn, which are all co-expressed in HeLa cells, takes place downstream of Cdk1 activation during M phase^18^. Furthermore, Src-family kinases, albeit small fractions, are definitely present in the nucleus^18^,^19^,^20^. To examine the effect of nuclear expression of Lyn, a member of the Src family, on progression of cells from G2 phase into M phase, Lyn was tagged with a nuclear localization signal at the amino-terminus (NLS-Lyn) and the tetracycline-inducible expression of NLS-Lyn or a kinase-dead mutant of NLS-Lyn [NLS-Lyn(KD)] was generated in HeLa S3 cells [HeLa S3/NLS-Lyn and HeLa S3/NLS-Lyn(KD) cell lines]. Western blotting analysis showed that NLS-Lyn and NLS-Lyn(KD) were inducibly expressed by Dox addition and that autophosphorylation, which leads to activation of the kinase activity, was detected on NLS-Lyn but not NLS-Lyn(KD) (Fig. 2a; Supplementary Fig. S1), confirming that NLS-Lyn(KD) is deficient in the kinase activity. Intriguingly, upon induction of NLS-Lyn by Dox treatment, HeLa S3/NLS-Lyn cells showed the increased number of cells exhibiting anaphase chromosome bridges and lagging chromosomes (Fig. 2b,c), indicative of chromosomal instability and characteristics of chromosome missegregation^33^,^34^,^35^. In contrast to NLS-Lyn, NLS-Lyn(KD) expression did not increase the frequency of chromosome missegregation (Fig. 2c). To further examine the role of the kinase activity of Src-family kinases in induction of chromosome missegregation, we used the Src kinase inhibitor PP2. In fact, treatment of NLS-Lyn-expressing cells with PP2 inhibited the induction of chromosome missegregation (Fig. 2d). These results suggest that induction of chromosome missegregation is dependent on the kinase activity of NLS-Lyn.

Then, we examined the period of time when the kinase activity of NLS-Lyn acts on the induction of chromosome missegregation. Since the appearance of H3pS10 begins in early prophase at the onset of mitotic chromosome condensation^36^, cells were found to enter prophase 11~12 h after release from thymidine-synchronized G1/S arrest (Fig. 2e). The kinase activity of Dox-induced NLS-Lyn was inhibited by the treatment with PP2 for (ii) 6 h, (iii) the last 3 h, or (iv) the last 1 h (Fig. 2f). Intriguingly, NLS-Lyn-induced chromosome missegregation was inhibited in any of these treatment times, indicating that treatment with PP2 for the last 1 h was sufficient for inhibiting the induction of chromosome missegregation (Fig. 2f; see also Fig. 2e). These results suggest that nuclear tyrosine kinase activities in prophase with the onset of chromosome condensation may be critical for exacerbation of chromosome missegregation.

### Attribution of chromosome missegregation to a lack of lipid modification of Lyn

**Figure 3:**
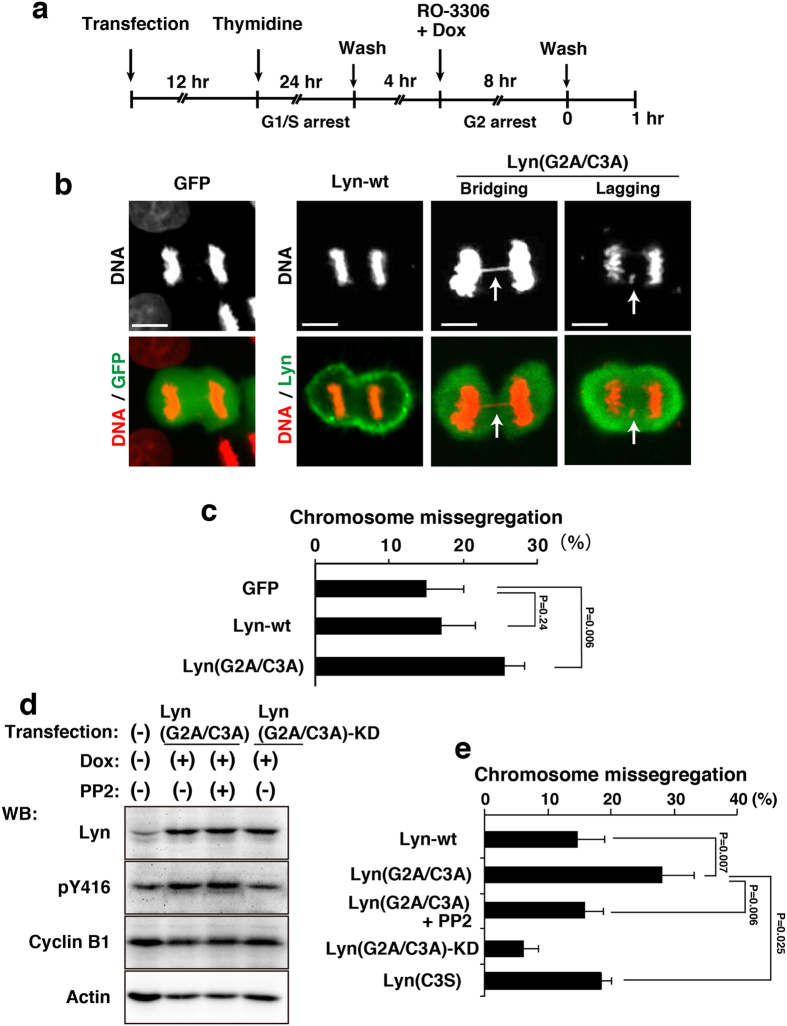
Chromosome missegregation induced by lack of lipid modifications in Lyn. HeLa S3/TR cells transiently transfected with GFP (control), Lyn-wt, or Lyn(G2A/C3A) were arrested at G1/S phase, released for 4 h, and further treated with 9 μM RO-3306 and 1 μg/ml Dox for 8 h. G2-arrested cells were washed, released into drug-free medium to enter M phase, and fixed after 1 h from the release. (**a**) Schematic depiction of cell synchronization. (**b**) Cells entering M phase were stained for GFP and Lyn proteins (green) and DNA (red). Representative images of cells are shown. Scale bars, 10 μm. (**c**) The percentages of cells exhibiting chromosome missegregation during anaphase and telophase were quantitated (>120 cells). Values are means ± S.D. from more than three independent experiments, and the significant difference is calculated by Student’s *t*-test. P = 0.24 indicates no significant difference. (**d**) HeLa S3/TR cells transiently transfected with Lyn(G2A/C3A) or Lyn(G2A/C3A)-KD were synchronized as shown in (**a**). The cells were treated with 10 μM PP2 for the last 1 h during 8 h-treatment with 9 μM RO-3306 and 1 μg/ml Dox, and then the released cells were further treated with 10 μM PP2 for 1 h. Western blotting was performed with anti-Lyn, anti-Src[pY^416^] (pY416), anti-cyclin B1, and anti-actin antibodies. A blot with anti-phospho-tyrosine (pTyr) antibody was shown in Supplementary Fig. S2b. Full-length blots are also presented in Supplementary Fig. S2. (**e**) The cells exhibiting chromosome missegregation (chromosome bridging and lagging) were quantitated (>82 cells). Values are means ± S.D. from more than three independent experiments, and significant differences are calculated by Student’s *t*-test.

Newly synthesized Lyn, in which the initiating methionine is removed by methionine aminopeptidase during translation, is myristoylated at glycine-2 by N-myristoyl transferase and subsequently palmitoylated at cysteine-3 by palmitoyl acyltransferase, and these lipid modifications are essential for membrane association of Src-family tyrosine kinases, including Lyn^5^,^6^,^10^. We used Lyn(G2A/C3A), a non-lipid modified Lyn by replacing glycine-2 and cysteine-3 with alanine residues, because the double alanine mutation of glycine-2 and cysteine-3 completely blocks membrane anchorage of Lyn, thereby increasing the amount of Lyn within the nucleus^19^. We then examined whether chromosome missegregation was induced by the expression of Lyn(G2A/C3A) in place of NLS-Lyn. To synchronize cells in M phase, HeLa S3/TR cells transiently expressing GFP (green fluorescent protein as a vector control), wild-type Lyn (Lyn-wt), or Lyn(G2A/C3A) were arrested at the G2/M boundary by treatment with RO-3306 for 8 h and released into drug-free medium for 60 min to enter M phase (Fig. 3a–c). Notably, the number of cells exhibiting chromosome missegregation was significantly increased upon expression of Lyn(G2A/C3A) compared to that of Lyn-wt or GFP (Fig. 3b,c). Western blotting analysis showed that strong autophosphorylation of Lyn(G2A/C3A) were detected during M phase (Fig. 3d; Supplementary Fig. S2a). Considering that attachment of the NLS sequence to the amino-terminus of Lyn not only promotes nuclear localization of Lyn but also blocks its amino-terminal lipid modifications, these results suggest that, like NLS-Lyn, a lack of lipid modifications of Lyn significantly increases the frequency of chromosome missegregation.

To examine the role of the kinase activity of Lyn(G2A/C3A) in induction of chromosome missegregation, we used PP2 and Lyn(G2A/C3A)-KD (kinase-dead). HeLa S3/TR cells transiently transfected with Lyn(G2A/C3A) were treated with Dox for inducible expression. Western blotting analysis showed that Dox-induced Lyn(G2A/C3A) was autophosphorylated in M phase (Fig. 3d) and that PP2 treatment decreased tyrosine phosphorylation levels of cellular proteins to some extent (Supplementary Figs S2b and S3). Treatment of Lyn(G2A/C3A)-expressing cells with PP2 significantly inhibited Lyn(G2A/C3A)-induced chromosome missegregation (Fig. 3e). Then, we confirmed by Western blotting analysis that Lyn(G2A/C3A)-KD is devoid of the kinase activity (Fig. 3d; Supplementary Fig. S2). It is of quite interest to note that expression of Lyn(G2A/C3A)-KD strongly inhibited chromosome missegregation (Fig. 3e), assuming that Lyn(G2A/C3A)-KD may act as a dominant-negative mutant form toward endogenous Lyn that fails in myristoylation and palmitoylation. On the other hand, Lyn(C3S), which can be myristoylated but not palmitoylated, did not exacerbate chromosome missegregation levels compared with Lyn(G2A/C3A). Furthermore, Lyn(G2A/C3A) was delocalized throughout the inside of a cell, whereas Lyn-wt was seen restrictedly at the plasma membrane in M phase (Fig. 3b). However, as the level of Lyn(G2A/C3A)-mediated tyrosine phosphorylation was not the highest among Lyn mutants (Supplementary Fig. S4), we consider that the levels of the kinase activity of Lyn mutants are not correlated with those of chromosome missegregation. These results suggest that non-membrane-bound Lyn, such as Lyn(G2A/C3A) and NLS-Lyn, is associated with the induction of chromosome missegregation during M phase.

### Effect of lipid modifications of c-Src and Lyn on induction of chromosome missegregation

**Figure 4:**
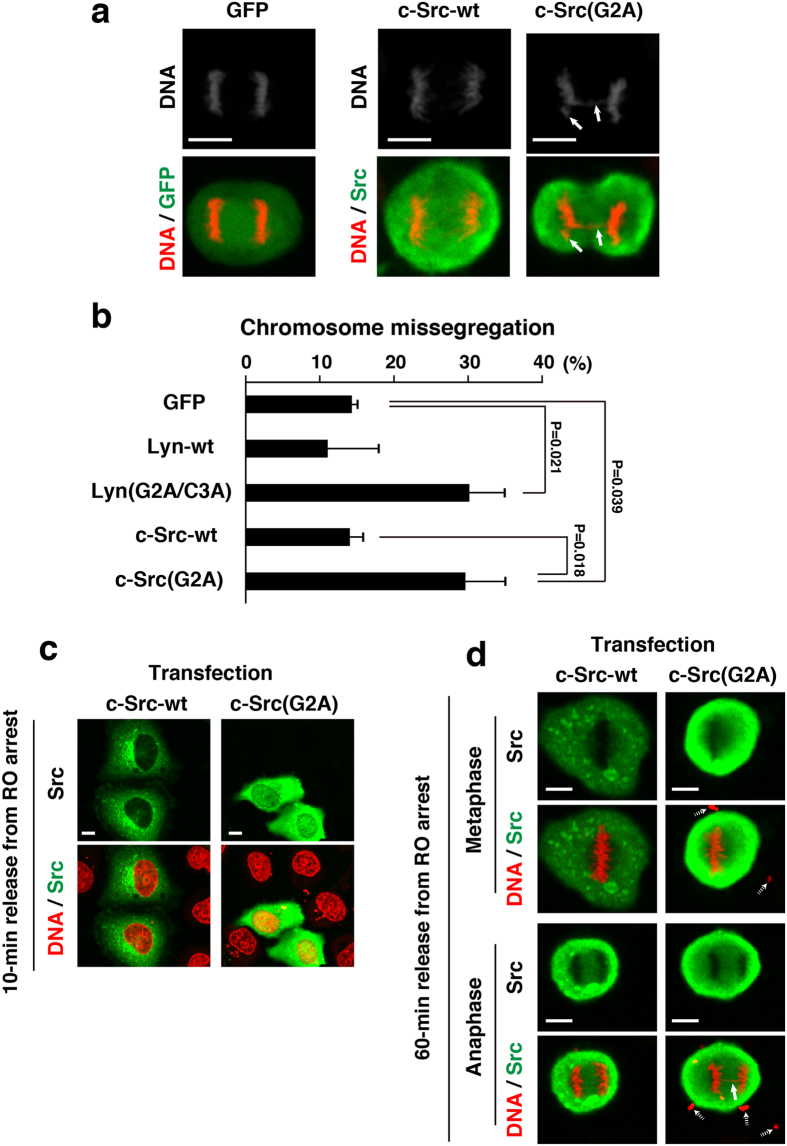
Protective role of Src myristoylation against chromosome missegregation. (**a**) HeLa S3/TR cells transiently transfected with GFP, c-Src, or c-Src(G2A) were synchronized as shown in Fig. 3a. Bold arrows indicate a lagging chromosome and a chromosome bridge. (**b**) The cells exhibiting chromosome missegregationn (chromosome bridging and lagging) were quantitated (>62 cells). Values are means ± S.D. from more than three independent experiments, and the significant differences are calculated by Student’s *t*-test. (**c**,**d**) HeLa S3/TR cells transiently transfected with c-Src or c-Src(G2A) were synchronized as shown in Fig. 3a, and were released from RO-3306-treated arrest at the G2/M boundary for 10 min (early prophase; see Fig. 1) (**c**) and 60 min (metaphase and anaphase) (**d**). Cells were stained for Src proteins (green) and DNA (red). A bold arrow indicates a chromosome bridge. Dotted arrows show plasmid DNA polyplexes deposited extracellularly, which were added to cell cultures for transient transfection. Note that plasmid DNA polyplexes were often observed in transient transfection cultures but not inducibly expression cultures.

Whereas Lyn-wt is in general doubly myristoylated and palmitoylated, wild-type c-Src (c-Src-wt) is myristoylated but not palmitoylated because of the absence of the palmitoylation site in the entire amino acid sequence of c-Src^5^,^6^,^10^. To examine whether the lack of myristoylation of c-Src resulted in exacerbating chromosome missegregation, we prepared c-Src(G2A), which lacks the myristoylation site resulting in production of non-lipid-modified c-Src. HeLa S3/TR cells transiently transfected with c-Src or c-Src(G2A) were synchronized as shown in Fig. 3a and compared the levels of chromosome missegregation between c-Src and c-Src(G2A) expressions. Expression of c-Src(G2A) increased the frequency of chromosome missegregation at a similar extent to that of Lyn(G2A/C3A) (Fig. 4a,b). Notably, c-Src(G2A) was delocalized to the cytoplasm and the nucleus in early prophase and to the cytoplasm in metaphase and anaphase, whereas c-Src-wt was largely localized to punctate structures (Fig. 4c,d). Considering that the level of c-Src(G2A)-mediated tyrosine phosphorylation was comparable to that of c-Src-wt (Supplementary Fig. S5), these results suggest that a lack of lipid modifications of Src-family kinases results in exacerbating chromosome missegregation owing to their delocalization. In other words, their membrane anchorage through lipid modifications from prophase to anaphase plays a protective role in induction of chromosome missegregation.

## Discussion

In the present study, we show that the defect in lipid modifications of Lyn and c-Src is significantly involved in induction of chromosome missegregation, such as formation of lagging chromosomes and anaphase chromosome bridges, in a kinase activity-dependent manner. Time course analysis with PP2 shows that the kinase activity of non-lipid-modified, non-membrane-bound Src during M phase is critical for giving rise to chromosome missegregation.

We have shown that the way of lipid modifications of Src-family kinases plays an important role in their intracellular membrane trafficking and nuclear translocation^7^,^10^,^13^,^19^. Lyn’s myristoylation at glycine-2 and its subsequent palmitoylation at cysteine-3 and c-Src’s myristoylation at glycine-2 are needed for their association with the plasma membrane^5^,^6^. In addition, Lyn mutants lacking the lipid modification sites are accumulated within the nucleus, suggesting that deletion of the lipid modification sites of Lyn enhances the accumulation of Lyn within the nucleus^19^. Mutation of glycine-2 within Gα subunits, despite the preservation of cysteine-3, was reported to prevent not only myristoylation but also palmitoylation^37^. However, the double alanine mutation of glycine-2 and cysteine-3 on Lyn fully ensures inhibition of its membrane anchoring, thereby increasing the amounts of nuclear localization of Lyn. Like Lyn(G2A/C3A), c-Src(G2A) is non-lipid-modified c-Src and is accumulated in the nucleus because wild-type c-Src has a mono-myristoylation site at glycine-2 but not any palmitoylation sites (Fig. 4). Notably, previous study showed that approximately 16% of newly synthesized v-Src and c-Src are normally not myristoylated and fail to anchor to membranes^38^. Indeed, we showed that Src-family members, c-Src, c-Yes, Lyn and Fyn, are endogenously present in the nucleus^17^,^19^,^20^. These results suggest that the failure in lipid modifications of endogenous Src-family kinases may result in their nuclear accumulation.

Not only c-Src but also Lyn, Fyn and c-Yes are all activated upon mitotic entry by Cdk1-cyclin B1 and/or PTPα^18^,^23^,^30^,^31^,^39^. Considering that expression of Lyn(G2A/C3A) results in the increased levels of chromosome missegregation similar to that of NLS-Lyn, which is incapable of being lipid modified owing to the addition of an NLS sequence at the N-terminus of Lyn (Figs 2c and 3e), we assume that induction of chromosome missegregation may be attributable to the presence of the kinase activity in the nucleoplasm during prophase and in the cytoplasm after nuclear envelope breakdown. Provided that anaphase chromosome bridging is caused by pre-mitotic defects, such as DNA double-strand breaks^40^ or mitotic defects in securin or cohesin degradation^41^,^42^, the presence of non-lipid-modified, non-membrane-bound Src-family kinases during M phase may bring about chromosome missegregation through their increased accessibility to mitotic chromosome-associated proteins.

Chromosomal instability, defined as frequent changes in chromosome structure and number, is increasingly appreciated as a key component of tumorigenesis^43^,^44^. The process of chromosomal instability involves chromosome missegregation at anaphase in some or most instances^45^. Anaphase chromosome bridges are associated with chromosome missegregation and have occasionally been observed in colorectal neoplasia and in sarcomas^46^. Furthermore, anaphase chromosome bridges have been strongly linked to chromosomal instability in human tumor samples^40^,^46^,^47^ and tumorigenesis in mice^48^. There is a strong correlation between anaphase bridges and multipolar mitoses, and the induction of dicentric chromosomes by gamma irradiation and telomerase inhibition leads to an elevated frequency of multipolar mitotic spindles, suggesting that multipolarity can result from polyploidization triggered by anaphase bridges. Moreover, anaphase bridges can arise from a variety of causes, including telomere dysfunction, cleavage defect of securin or cohesion^41^,^42^, or decatenation failure. Given that an excess amount of non-lipid-modified Src-family kinases impairs correct chromosome segregation, lipid modifications of Src-family kinases may be physically confined to their proper membrane-associated localization during mitosis, leading to prevent chromosome missegregation. Thus, a limited level of tyrosine phosphorylation during mitosis by non-membrane-bound Src-family kinases may be important for proper processing of chromosome segregation.

In conclusion, we show that mitotic expression of non-lipid-modified Src-family kinases significantly induces chromosome missegregation. Membrane association of Src-family kinases plays a protective role against chromosome missegregation. Further studies will help us to understand the mechanism of Src-induced chromosome missegregation.

## Methods

### Plasmids, transfection, and cells

Lyn-wt (wild-type Lyn), Lyn(G2A/C3A) [glycine at position 2 (glycine-2) → alanine, cysteine at position 3 (cysteine-3) → alanine], Lyn(G2A/C3A)-KD [Lyn(G2A/C3A/K275A)] (glycine-2 → alanine, cysteine-3 → alanine; kinase-dead, lysine at position 275 → alanine), Lyn(C3S) (cysteine-3 → serine), NLS-Lyn (nuclear localization signal-tagged Lyn), and NLS-Lyn(KD) [NLS-Lyn(kinase-dead)] (nuclear localization signal-tagged Lyn; kinase-dead, lysine at position 275 → arginine, tyrosine at position 508 → phenylalanine) were constructed from cDNA coding for human wild-type Lyn^49^ (provided by T. Yamamoto), as described previously^7^,^10^,^13^,^18^,^19^,^21^. For inducible protein expression, all constructs were subcloned into the pcDNA4/TO vector (Invitrogen)^50^. pcDNA4/TO/FH-NLS-Lyn-wt (FLAG- and HA-tagged, nuclear localization signal-tagged, wild-type Lyn) and pcDNA4/TO/FH-NLS-Lyn(KD) [FH-tagged NLS-Lyn(kinase-dead)] were generated as follows. The FH-NLS-Lyn-wt fragment was created by PCR using Lyn-wt or Lyn(KD) as a template with the sense primer 5′-ATAGAATTCCGTTGAACCATGGACTACAAGGACGACGATGACAAGC-3′ and the antisense primer 5′-GGAGCGGCCGCCCTGTGCTCTSSGGCTGCTGCTG-3′. The FH-NLS-Lyn(KD) fragment was designed by PCR using the sense primer 5′-ATAGAATTCCGTTGAACCATGGACTACAAGGACGACGATGACAAGC-3′ and the antisense primer 5′-GGAGCGGCCGCCCTGTGCTCTSSGGCTGCTGCTGGAATTGCCCTTCCGT-3′. The *EcoR*I–*Not*I fragment of the PCR product was introduced into the *EcoR*I–*Not*I sites of pcDNA4/TOneo^50^. Green fluorescent protein (GFP) was obtained from the pEGFP-C1 vector (Clontech Laboratories, Inc.) and subcloned into pcDNA4/TO^51^. v-Src-wt and v-Src(K295M) (kinase-dead, lysine at position 295 → methionine)^52^ (provided by H. Ohnishi) were subcloned into pcDNA4/TO^29^. c-Src-HA and c-Src(G2A)-HA were constructed from pcDNA3/c-Src-HA^53^ (provided by S.A. Laporte). pcDNA4/TO/c-Src-HA was constructed as described previously^27^. The glycine → alanine mutation at position 2 in c-Src(G2A)-HA was created by site-directed mutagenesis with c-Src-HA as a template using the sense primer 5′-GTGGAATTCCGACCACCATGGCTAGCAACAAGAGCAAGCCCAAGGATGC-3′ and the antisense primer 5′-CTTGGGCTTGCTCTTGTTGCTAGCCATGGTGGTCGGAATTCCACCACAC-3′. The *EcoR*I–*Xba*1 fragment of the PCR product was introduced into the *EcoR*I–*Xba*1 sites of pcDNA4/TO/neo^27^. Transient and stable transfection was performed using 25-kDa linear polyethylenimine (Polysciences, Inc.)^54^. To establish stable cell lines that inducibly express either NLS-Lyn or NLS-Lyn(KD), HeLa S3 cells (Japanese Collection of Research Bioresources, Osaka, Japan) were co-transfected with the pCAG vector encoding tetracycline repressor (TR)^51^ and a plasmid encoding the hygromycin resistance gene, and selected in 200 μg/ml hygromycin (HeLa S3/NLS-Lyn, HeLa S3/NLS-Lyn(KD)). Cells stably expressing TR (HeLa S3/TR) were transfected with pcDNA4/TO/NLS-Lyn or pcDNA4/TO/NLS-Lyn(KD), and cell clones inducibly expressing NLS-Lyn or NLS-Lyn(KD) were selected in 500 μg/ml G418.

### Antibodies

The following antibodies are used: Lyn (clone 9, Wako; H-6, sc-7274, Santa Cruz Biotechnology), actin (C4, Millipore; 13E5, Cell Signaling Technology), phosphotyrosine (pTyr) (4G10, Upstate Biotechnology Inc.), Src (#327, Calbiochem), anti-Src[pY^416^] (phospho-Src family, Cell Signaling Technology), cyclin B1 (H-433, Santa Cruz Biotechnology; V152, Cell Signaling Technology), phospho-histone H3 serine at position 10 (H3pS10) (clone 6G3, #9706 S, Cell Signaling Technology), and α-tubulin (MCA78G, Serotec). Horseradish peroxidase (HRP)-F(ab′)_2_ fragments of anti-mouse IgG antibody, anti-rabbit IgG antibody, and of anti-rat IgG antibody, and Alexa Fluor 488-donkey-anti-mouse IgG antibody were purchased from GE Healthcare, Cell LAB, and Invitrogen.

### Immunofluorescence microscopy

Immunofluorescence staining was performed as described^11^,^29^,^55^. In brief, cells were fixed with 4% paraformaldehyde in phosphate-buffered saline (PBS) for 20 min, and permeabilized with 0.2% Triton X-100 in PBS for 5 min. Moreover, fixed cells were blocked with 3% bovine serum albumin in PBS containing 0.1% saponin, and then sequentially incubated with a primary and a secondary antibody for 1 h each. DNA was stained with 20 μg/ml propidium iodide or 20 nM TO-PRO-3 (Molecular Probes) for 30 min after treatment with 200 μg/ml RNase A. Confocal and Nomarski differential-interference-contrast (DIC) images were obtained using a Fluoview Fv500 laser scanning microscope with 40 × 0.95 N.A. objective (Olympus, Tokyo, Japan). Composite figures were prepared using Photoshop 13.0 and Illustrator 16.0 software (Adobe). Note that permeabilization of paraformaldehyde-fixed metaphase cells with 0.2% Triton X-100 was prone to reduce the intensity of c-Src-wt due to an unknown reason (see Fig. 4d).

### Flow cytometry

Flow cytometric analysis was performed as described^27^,^56^,^57^. In brief, cells detached by trypsinization were fixed in 4% paraformaldehyde for 1 h, permeabilized with 70% ethanol for at least 1 h at −30 °C, and blocked with 3% bovine serum albumin in PBS containing 0.1% saponin for 30 min at room temperature. After washing with PBS containing 0.1% Tween 20, cells were co-immunostained with anti-H3pS10 and anti-cyclin B1 antibodies for 1 h at room temperature, then stained with secondary antibodies. DNA staining was done by propidium iodide after treatment with RNase A. A minimum of 5,000 cells per sample was analyzed by flow cytometry using a Guava easyCyte (Millipore) equipped with a 488-nm laser and a 640-nm laser using Flowing Software version 2.5.0 (Perttu Terho, Centre for Biotechnology, Turku, Finland). Cell debris was excluded by gating on forward scatter and pulse-width profiles.

### Western blotting

Cells were lysed in SDS-sample buffer, and cell lysates were subjected to SDS-PAGE and transferred onto polyvinylidene difluoride membranes (Millipore). To detect tyrosine phosphorylation of proteins, cell lysates were prepared with SDS-sample buffer containing 10 mM sodium orthovanadate, 20 mM ß-glycerophosphate, and 50 mM NaF. Immunodetection was performed by enhanced chemiluminescence and examined using an image analyzer ChemiDoc XRSplus (Bio-Rad) as reported previously^11^,^50^. Sequential reprobing of membranes with various antibodies was performed after the removal of antibodies from the membranes or inactivation of HRP by 0.1% NaN_3_, according to the manufacturer’s instructions.

### Cell cycle synchronization

HeLa S3/NLS-Lyn or HeLa S3/NLS-Lyn(KD) cells were arrested in G1/S phase with 4 mM thymidine for 24 h. After 6-h release from G1/S-phase arrest in thymidine-free fresh medium, the cells were incubated for further 6 h in medium with or without 1 μg/ml doxycycline (Dox), a tetracycline derivative, for inducible expression. Cells were treated with the Src inhibitor PP2 (10 μM) and Dox from the last 3 h incubation (Fig. 2d). HeLa S3/TR cells were treated with 4 mM thymidine for 24 h and arrested at G1/S phase (Fig. 2a). Subsequently, the cells were released for 4 h. To synchronize cells in G2 phase, cells were treated with the cyclin-dependent kinase 1 (Cdk1) inhibitor RO-3306 (9 μM) (Calbiochem) for 8 h with/without Dox (1 ng/ml~2 μg/ml). Cells arrested at the G2/M boundary were washed with PBS containing Ca^2+^ and Mg^2+^ [PBS(+)], released into M phase, and fixed at 1 h from the release^56^. Cells were treated with 10 μM PP2 for the last 1-h incubation of RO-3306 and Dox (Fig. 4). Double thymidine block was performed as described previously^50^,^57^. Cells treated with 4 mM thymidine for 15 h were incubated in complete medium without thymidine for 9 h, and the cells were further treated with 4 mM thymidine for 15 h (Fig. 1).

## Additional Information

**How to cite this article**: Honda, T. *et al*. Protective role for lipid modifications of Src-family kinases against chromosome missegregation. *Sci. Rep.* **6**, 38751; doi: 10.1038/srep38751 (2016).

**Publisher's note:** Springer Nature remains neutral with regard to jurisdictional claims in published maps and institutional affiliations.

## Supplementary Material

Supplementary Information
