# Supplementary material for: Protective role for lipid modifications of Src-family kinases against chromosome missegregation
Source: Sci Rep. 2016 Dec 12;6:38751. doi: 10.1038/srep38751 (PMC5150256; doi:10.1038/srep38751)
Supplement: Supplementary Information [file srep38751-s1.pdf]

Supplementary Information

Protective role for lipid modifications of Src-family kinases against chromosome missegregation

Takuya Honda[1,3], Shuhei Soeda[1,3], Kunihiko Tsuda[1], Chihiro Yamaguchi[1], Kazumasa Aoyama[1], Takao Morinaga[1], Ryuzaburo Yuki[1], Yuji Nakayama[1, 2], Noritaka Yamaguchi[1], and Naoto Yamaguchi[1, *]

[1]Laboratory of Molecular Cell Biology, Graduate School of Pharmaceutical Sciences, Chiba University, Chiba 260-8675, Japan

[2]Department of Biochemistry and Molecular Biology, Kyoto Pharmaceutical University, Kyoto 607-8414, Japan

*Corresponding author: Naoto Yamaguchi, Ph.D.

Laboratory of Molecular Cell Biology, Graduate School of Pharmaceutical Sciences, Chiba University, Inohana 1-8-1, Chuo-ku, Chiba 260-8675, Japan

Tel & Fax: +81-43-226-2868

Email: nyama@faculty.chiba-u.jp

[3]These authors contributed equally to this work.

Supplementary Figures (Supplementary Fig. S1 ~ Fig. S5)

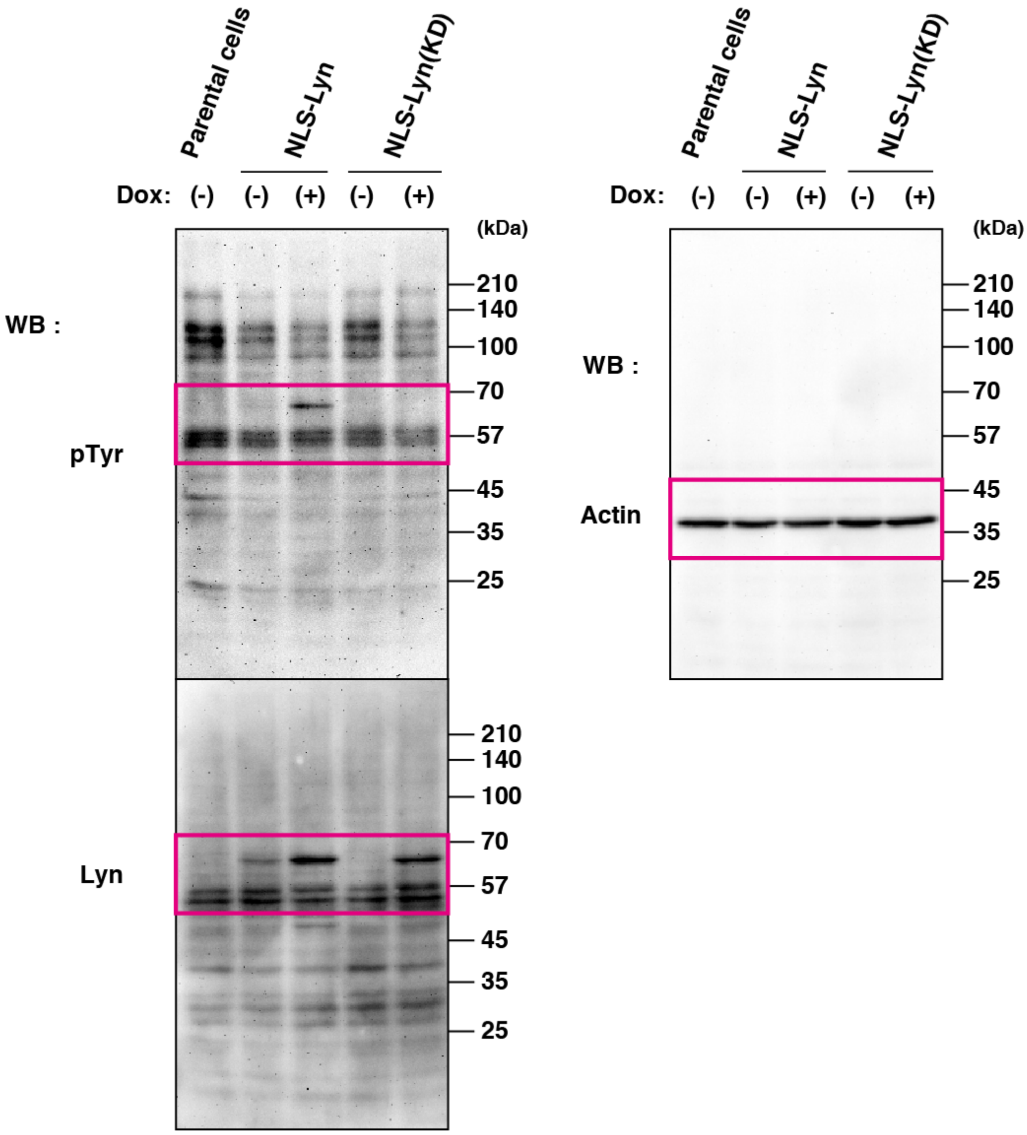

Supplementary Figure S1. Full-length blots for Fig. 2a.

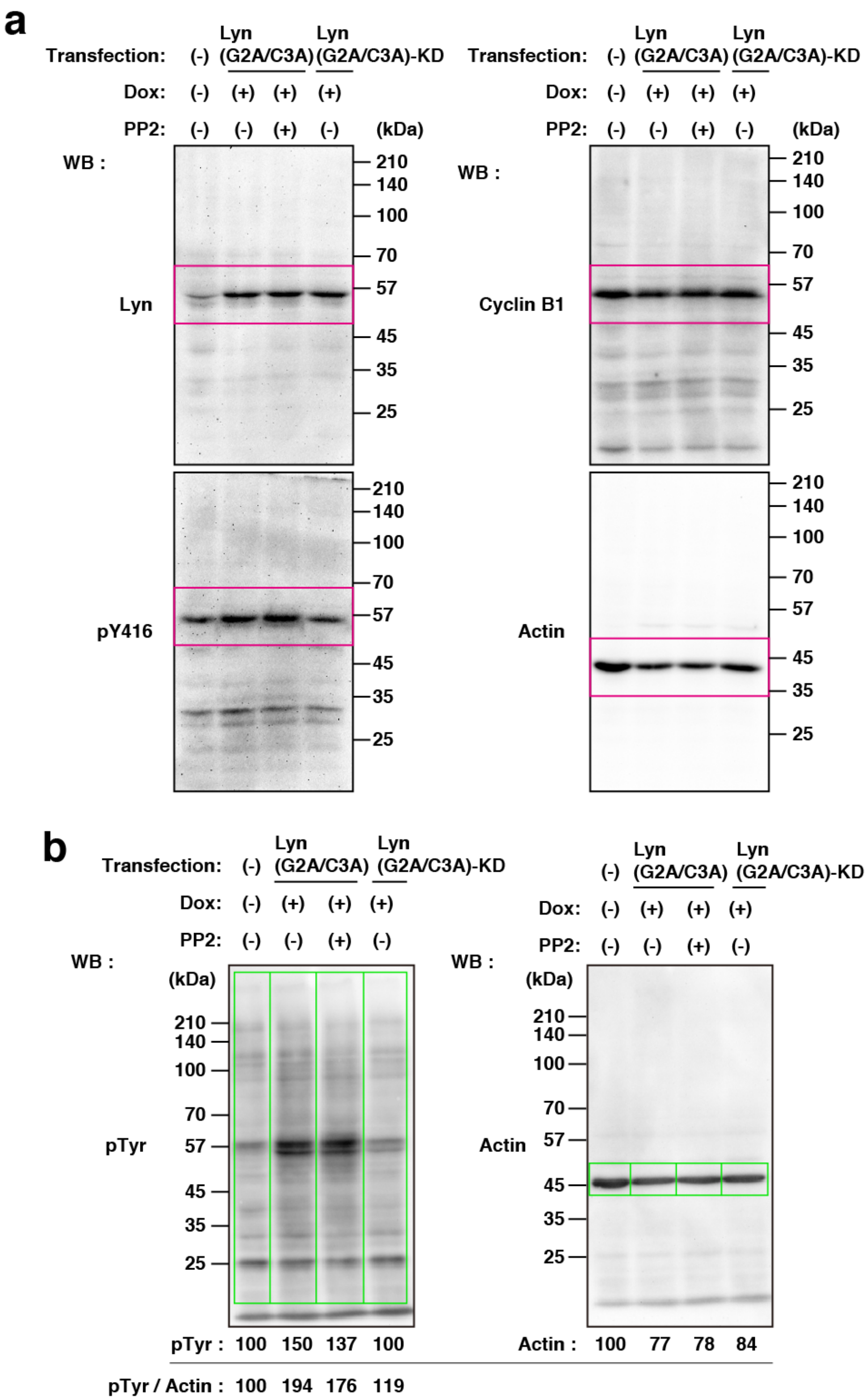

Supplementary Figure S2. Full-length blots for Fig. 3d.
(a) Full-length blots for Fig. 3d. **(b)** Tyrosine phosphorylation levels. The gel blotted with anti-pTyr and anti-actin antibodies has been run under the same experimental conditions for Fig. 3d. Levels of tyrosine phosphorylation of cellular proteins (boxed areas in left panel) and amounts of actin (boxed areas in right panel) were determined with sequential probing, and relative levels of tyrosine phosphorylation (pTyr/actin) were expressed at the bottom.

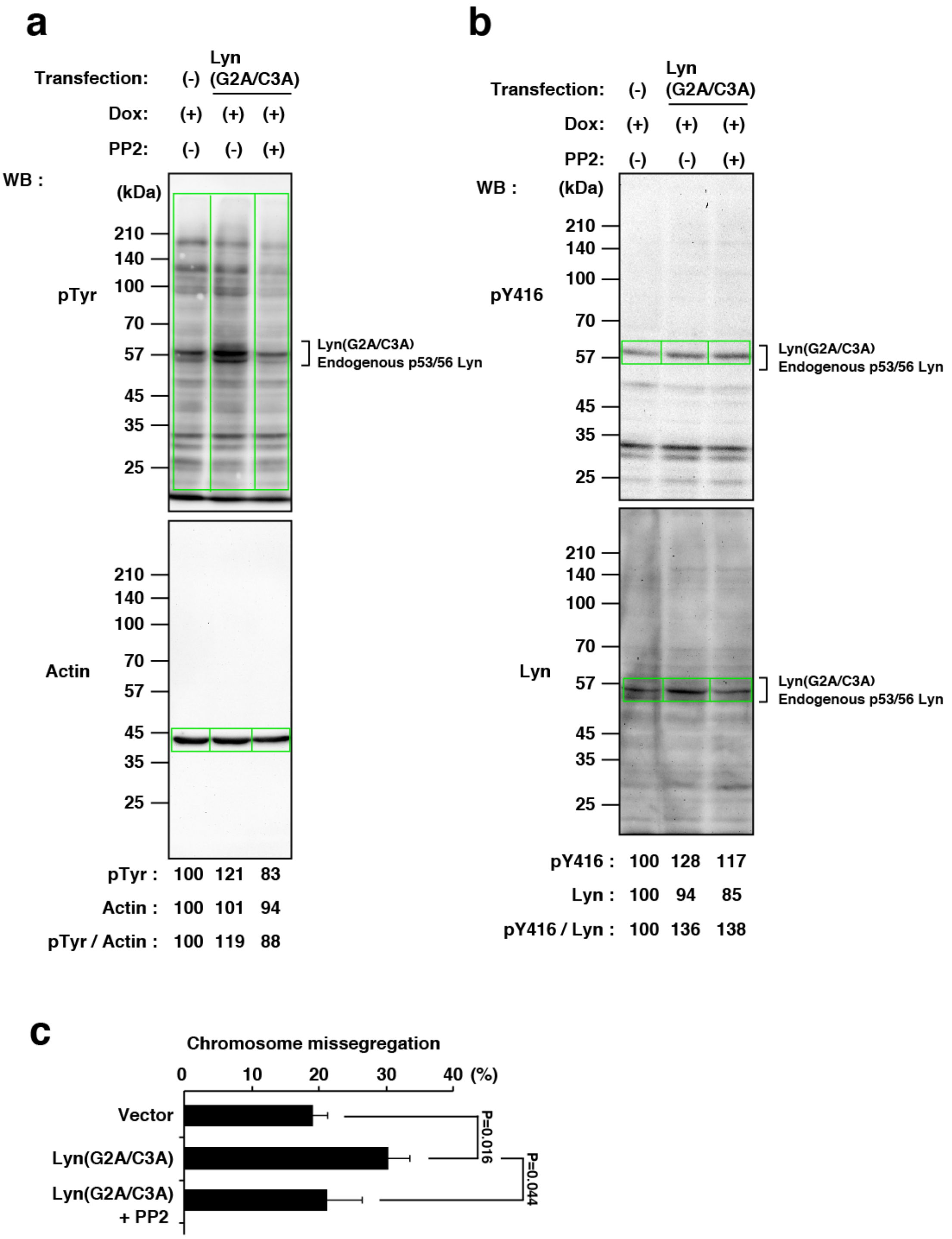

Supplementary Figure S3. Autophosphorylation and kinase activity.

HeLa S3/TR cells transiently transfected with Lyn(G2A/C3A) were synchronized as shown in Fig. 3a. The cells were treated with 20 μM PP2 throughout the last 9 h instead of the last 2 h (see Fig. 3d and Supplementary Fig. S2b). Western blotting was performed with **(a)** anti-pTyr and anti-actin antibodies and **(b)** anti-Src[pY^{416}] and anti-Lyn antibodies on two sets of blots. Relative levels of tyrosine phosphorylation (boxed areas in left panels, pTyr/actin) and relative levels of autophosphorylation (pY416) (right panels, pY416/Lyn) were expressed at the bottom. Note that PP2 treatment even for 9 h did not affect the autophosphorylation level despite a decrease in tyrosine phosphorylation levels of cellular proteins. **(c)** After treatment with PP2 as shown in (a, b), the cells exhibiting chromosome missegregation (chromosome bridging and lagging) were quantitated (>17 cells). Values are means ± S.D. from three independent experiments, and the significant differences are calculated by Student's *t*-test.

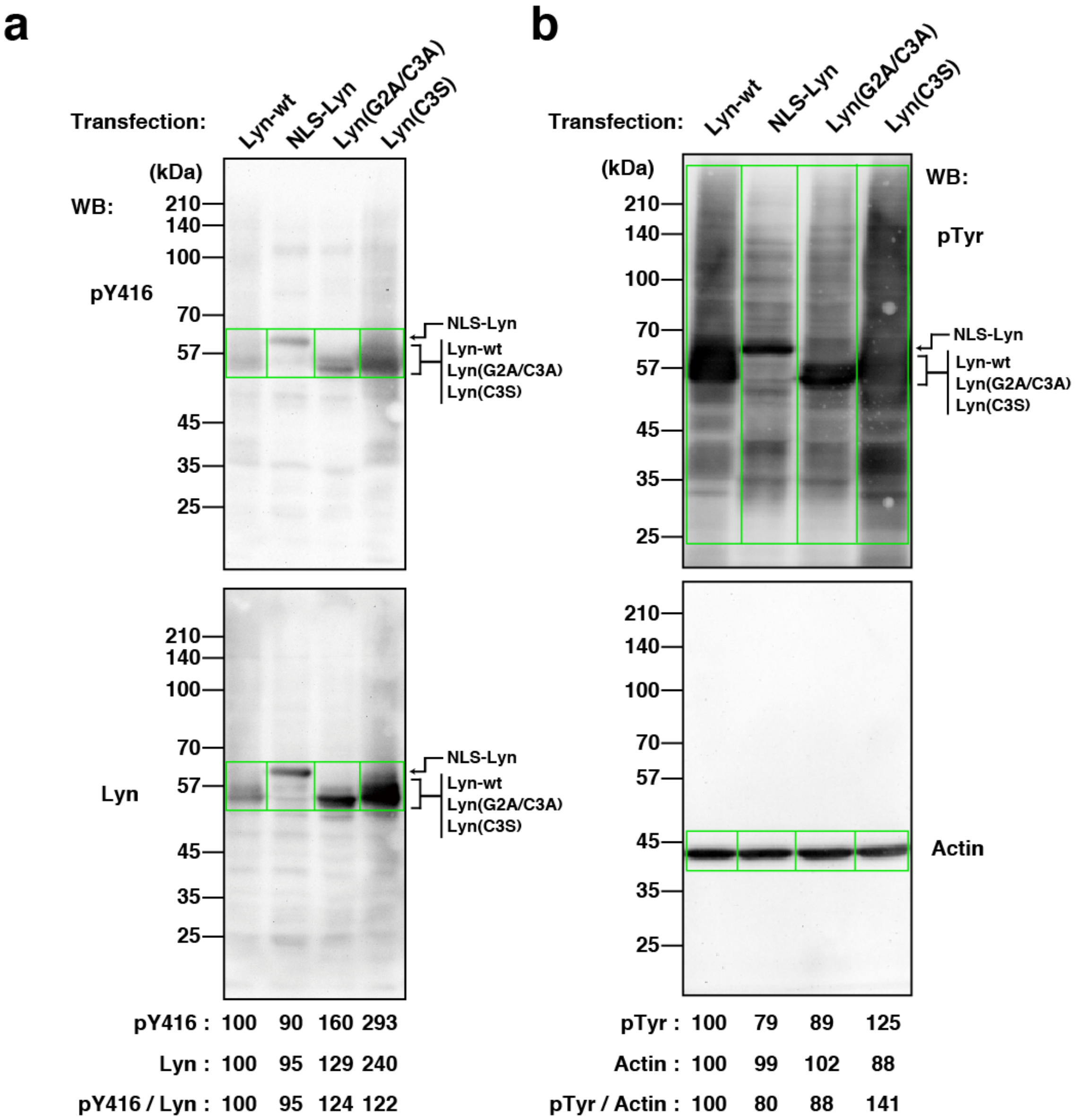

Supplementary Figure S4. Comparison of the kinase activities among Lyn and its mutants. COS-1 cells were transiently transfected with Lyn-wt, NLS-Lyn, Lyn(G2A/C3A), and Lyn(C3S) for 24 h. Western blotting was performed with **(a)** anti-Src[pY^{416}] (pY416) and anti-Lyn antibodies and **(b)** anti-pTyr and anti-actin antibodies on two sets of blots. Relative levels of autophosphorylation (boxed areas in left panels, pY416/Lyn) and relative levels of tyrosine phosphorylation (boxed areas in right panels, pTyr/actin) were expressed at the bottom.

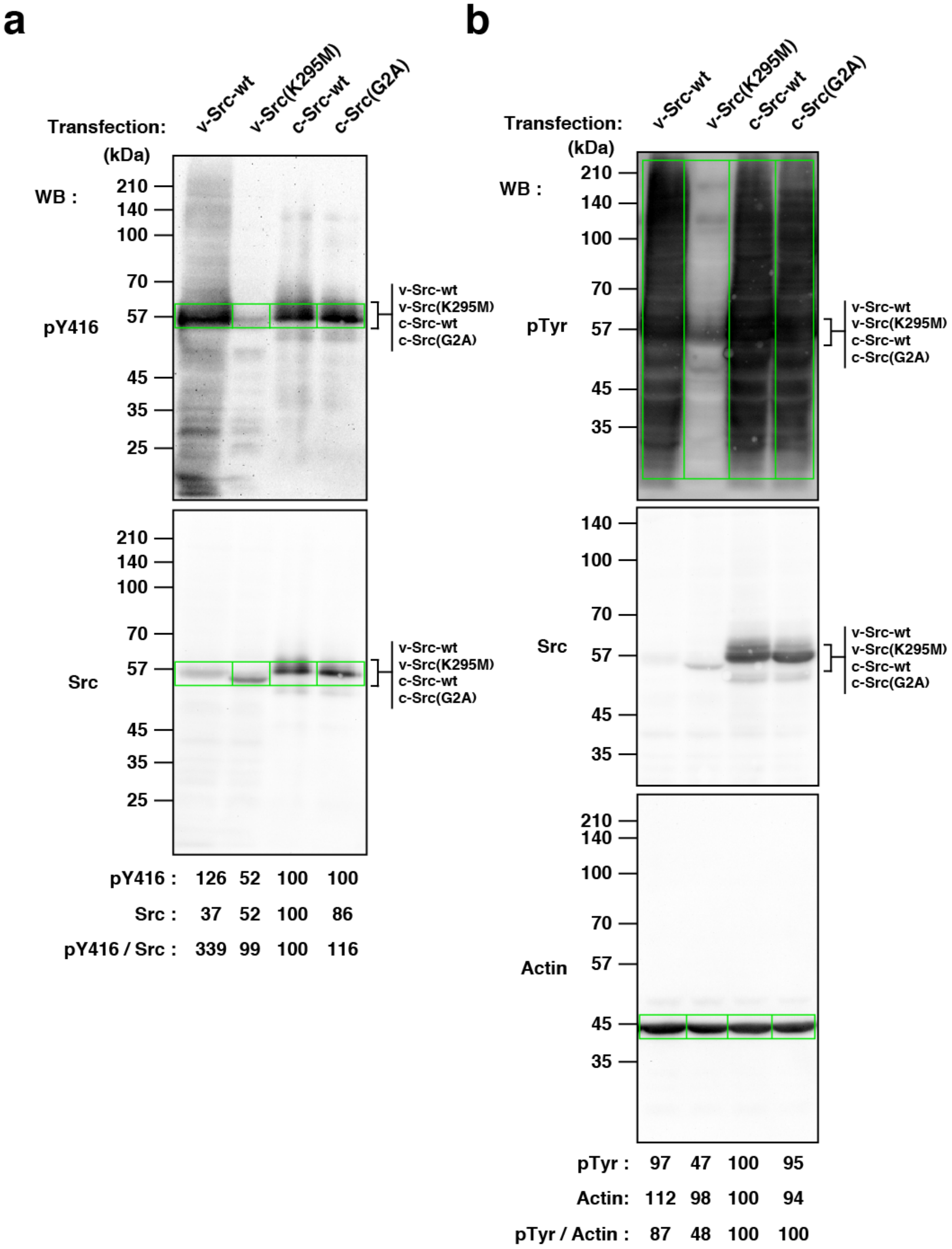

Supplementary Figure S5. Comparison of the kinase activities among Src and its mutants. COS-1 cells were transiently transfected with v-Src-wt, v-Src(K295M), c-Src-wt, and c-Src(G2A) for 24 h. Western blotting was performed with **(a)** anti-Src[pY^{416}] (pY416) and anti-Src antibodies and **(b)** anti-pTyr, anti-Src, and anti-actin antibodies on two sets of blots. Relative levels of autophosphorylation (boxed areas in left panels, pY416/Src) and relative levels of tyrosine phosphorylation (right panels, pTyr/actin) were expressed at the bottom. Note that the cell lysates somehow yielded only extremely small amounts of v-Src-wt and v-Src(K295M).
